# To win the battle: the chloroplast is a key battleground in plant–pathogen interactions

**DOI:** 10.1093/hr/uhaf294

**Published:** 2025-11-03

**Authors:** Lu Rui, Zhujiang Cong, Xinghuang Zhou, Qing Yang, Zhanchun Wang, Wei Wang

**Affiliations:** College of Biology and Food Engineering, Chongqing Three Gorges University, Chongqing 404120, China; College of Biology and Food Engineering, Chongqing Three Gorges University, Chongqing 404120, China; College of Biology and Food Engineering, Chongqing Three Gorges University, Chongqing 404120, China; College of Biology and Food Engineering, Chongqing Three Gorges University, Chongqing 404120, China; State Key Laboratory of Agricultural and Forestry Biosecurity, Key Laboratory of Ministry of Education for Genetics, Breeding and Multiple Utilization of Crops, Plant Immunity Center, Fujian Agriculture and Forestry University, Fuzhou 350002, China; State Key Laboratory of Agricultural and Forestry Biosecurity, Key Laboratory of Ministry of Education for Genetics, Breeding and Multiple Utilization of Crops, Plant Immunity Center, Fujian Agriculture and Forestry University, Fuzhou 350002, China

## Abstract

The interaction between plants and pathogens represents a complex evolutionary arms race. Plants employ a sophisticated innate immune system to combat pathogen invasion. However, pathogens inhibit plant immunity by secreting effectors into the host cell. The chloroplast is an indispensable organelle for photosynthesis and metabolism in plants. Notably, increasing evidence has recently revealed the pivotal role of chloroplasts in plant immunity, including reactive oxygen species production, phytohormone biosynthesis, and signal transduction. Accordingly, chloroplasts have emerged as key targets for pathogen effectors. In this review, we summarize the role of chloroplasts in plant immunity and update the identification of pathogen effectors that enhance pathogenicity by targeting chloroplasts. We also discuss the diverse mechanisms by which pathogen effectors hijack chloroplasts to manipulate plant immunity, shedding light on the functional complexity and importance of chloroplasts in plant–pathogen interactions.

## Introduction

The ongoing conflict between plants and pathogens has led to a continuing evolutionary arms race. To defend against pathogens, plants employ multiple immune receptors to recognize nonself organisms and initiate downstream responses. First, cell surface-localized pattern recognition receptors (PRRs), consisting of receptor-like kinases (RLKs) or receptor-like proteins (RLPs), perceive microbe- or pathogen-associated molecular patterns (MAMPs/PAMPs) and subsequently activate pattern-triggered immunity (PTI) [1]. PTI responses often include the activation of the mitogen-activated protein kinase (MAPK) cascade, the influx of calcium ions (Ca^2+^), and the burst of reactive oxygen species (ROS), which are triggered by PRR-phosphorylated receptor-like cytoplasmic kinases (RLCKs) and aim to restrict pathogen growth and pathogenicity [2].

To successfully inhibit PTI and invade plants, many pathogens, in turn, deliver effectors into the apoplasts or plant cells. Furthermore, plants employ intracellular nucleotide-binding (NB) and leucine-rich repeat (LRR) receptors (NLRs) to recognize effectors and initiate effector-triggered immunity (ETI), the second layer of plant innate immunity often accompanied by programmed cell death (PCD) or a hypersensitivity response (HR). Recent studies have revealed the intricate interplay and mutual reinforcement between PTI and ETI [1], which underscores the highly sophisticated defense mechanisms by which plants cope with a range of pathogens.

In addition, increasing evidence indicates that plant organelles play important roles in immunity, among which chloroplasts, essential organelles that are primarily responsible for photosynthesis, have emerged as pivotal players in this continuous struggle [3]. The chloroplast serves as the major position for the biosynthesis of phytohormones critical to immunity, such as salicylic acid (SA), jasmonic acid (JA), auxin (IAA), and gibberellic acid (GA), as well as their precursors. Additionally, chloroplasts generate ROS to combat pathogens and communicate with the nucleus, triggering the transcriptional reprogramming of nuclear-encoded chloroplast-targeted genes (*NECGs*). Moreover, it has been reported that many chloroplast-related proteins are involved in immunity. For instance, phosphorylation of the light-harvesting complex II component OsLHCB5 confers broad-spectrum blast resistance in rice [4]. GOLDEN2-LIKE 1 (GLK1) and GLK2, two transcription factors that regulate chloroplast development in *Arabidopsis*, contribute to defense against the pathogen *Hyaloperonospora arabidopsidis* in a JA-dependent manner [5].

Owing to their strategic importance, chloroplasts have been hotspot targets of pathogen effectors. For example, ectopic expression of the effectors AvrBs1 or AvrBs3 from *Xanthomonas campestris* pv. *vesicatoria* decreases the starch content in chloroplasts [6], suggesting that AvrBs1 and AvrBs3 may inhibit immunity by interfering with chloroplast carbon metabolism (e.g. starch synthesis), but their specific target proteins and molecular pathways remain unclear, representing important directions for future research. The movement protein TRIPLE GENE BLOCK2 (TGB2) of potato mop-top virus (PMTV) translocates to the chloroplast, associates with lipids, and disturbs the chloroplast ultrastructure [7]. Moreover, the chloroplast-localized effector C4 (cC4) from the tomato golden mosaic virus (TGMV) facilitates the recruitment of PLANT U-BOX 4 (PUB4), an E3 ligase, to the chloroplast outer membrane, thereby inducing chloroplast degradation and subsequently suppressing chloroplast-mediated immunity [8]. Recently, pathogen effectors that target chloroplasts have been increasingly identified via large-scale screens (Table 1). In this review, we update the identified effectors that are involved in the manipulation of immunity by targeting chloroplasts and summarize the strategies employed by pathogens to hijack chloroplasts, shedding light on this critical chloroplast battleground in plant–pathogen interactions.

**Table 1:** Summary of the effectors that affect chloroplast functions

No.	Effectors	Pathogen species	Plant species	Target proteins	Localizations	Mechanisms	References
1	AvrBs1	*X. campestris* pv. *vesicatoria*	*A. thaliana*	Unknown	Nucleus	Unknown	[6]
2	AvrBs3				Cytoplasm		
3	TGB2	Potato mop-top virus	Potato	Unknown	Chloroplasts	Disturbs the ultrastructural of chloroplast	[7]
4	C4	Tomato golden mosaic virus	Tomato	PUB4	Chloroplasts, plasma membranes	Degrades chloroplasts, degrades CERK1	[8]
5	HopK1	*P. syringae* pv. *tomato* DC3000	*A. thaliana*	Unknown	Chloroplasts	Unknown	[34]
6	AvrRps4				Chloroplasts, nucleus, cytoplasm		
7	CSEP080	*E. necator*	Grapevine	VviB6f	Chloroplasts, plasma membranes	Affects photosynthesis, disturbs cROS production	[10]
8	Pst_4	*P. striiformis* f. sp. *tritici*	Wheat	Ta(Sl)ISP	Cytoplasm	Suppresses import of host Fe–S protein into chloroplasts	[13]
9	Pst_5						
10	Pst_12806				Chloroplasts	Affects photosynthesis, disturbs cROS production	[12]
11	FolSvp2	*F. oxysporum* f. sp*. lycopersici*	Tomato		Chloroplasts		[11]
12	RXLR31154	*P. viticola*	Grapevine	PsbP	Chloroplasts	Reduces H_2_O_2_ accumulation, activates the ^1^O_2_ signaling pathway	[14]
13	HopN1	*P. syringae* pv. *tomato DC3000*	*N. benthamiana*	PsbQ	Chloroplasts	Degrades PsbQ, compromises cROS production	[15]
14	Fg03600	*F. graminearum*	Wheat	TaPGRL1	Chloroplasts	Suppresses cyclic photosynthetic electron flow	[16]
15	Pi22922	*P. infestans*	Potato	StFC-II	Chloroplasts	Disturbs heme and chlorophyll content balance, affects cROS	[17]
16	PsAvh113	*P. sojae*	Soybean	GmDPB	Cytoplasm	Degrades GmDPB, suppresses *CATALASE* transcription	[18]
17	AvrPiz-t	*M. oryzae*	Rice	CatB	Plasma membrane^b^	Activates ROS-scavenging cascade	[24]
18	P31	Maize chlorotic mottle virus	Maize	ZmCAT1	Cytoplasm	Inhibits catalase activity	[19]
19	HCPro	Chili veinal mottle virus	Chili, *N. tabacum*	CAT1, CAT3		Inhibits catalase activity	[20]
20	PpE18	*Phytophthora parasitica*	*N. benthamiana*	NbAPX3–1	Peroxisome membrane	Suppresses ascorbate peroxidase activity	[21]
21	AGH17488	*Candidatus liberibacter asiaticus*	*N. benthamiana*, *Clonorchis sinensis*	APX6	Chloroplasts	Reduces ascorbate peroxidase activity	[23]
22	PstGSRE4	*P. striiformis* f. sp. *tritici*	Wheat	TaCZSOD2	Unknown	Inhibits copper zinc superoxide dismutase activity	[22]
23	Gr-VAP1	*Heterodera schachtii*	*A. thaliana*	Unknown	Chloroplasts	Affects NPQ	[25]
24	γb	Barley stripe mosaic virus	Barley	NTRC	Chloroplasts	Impairs plant antioxidant defenses	[28]
25	PvCRN20	*P. viticola*	Grapevine	VvDEG5	Cytoplasm	Represses the import of VvDEG5 into chloroplasts, suppresses cROS	[30]
26	AvrXccC	*X. campestris* pv. *campestris*	*A. thaliana*	LSU1	Cytoplasm	Precludes relocalization of LSU1 to chloroplasts, affects cROS	[29]
27	AvrB2	*P. syringae* pv*. phaseolica*					

**Table 1:** Continued

No.	Effectors	Pathogen species	Plant species	Target proteins	Localizations	Mechanisms	References
28	HopR1	*P. syringae*					
29	EqCSEP01276	*E. quercicola*	Rubber tree	HbNCED5	Chloroplasts	Disturbs the localization of HbNCED5, inhibits ABA biosynthesis	[31]
30	HopI1	*P. syringae*	*A. thaliana*	Hsp70	Chloroplasts	Translocates its target protein into chloroplasts	[32]
31	AG1IA_06325	*R. solani*	Rice	OsV4	Chloroplasts	Affects development of chloroplasts	[35, 36]
32	AG1IA_07490						
33	AVRvnt1	*P. infestans*	Potato	GLYK	Chloroplasts	Mediates the GLYK degradation	[37]
34	Pt9029	*P. triticina*	Wheat	TaRCA	Chloroplasts	Decreases Rubisco enzyme activity, suppresses cROS production	[38]
35	AvrPik-D	*M. oryzae*	Rice	OsRBCS4	Chloroplasts		[39]
36	Pi22926	*P. infestans*	Potato	StTuB	Cytoplasm	Attenuates phosphorylation, reduces translocation	[40]
37	PsIsc1	*P. sojae*	Soybean	Unknown	Cytoplasm	Disrupts the plant salicylate metabolism pathway	[41]
38	VdIsc1	*V. dahliae*					
39	MoCHT1	*M. oryzae*	Rice	OsLLB	Chloroplasts	Suppresses JA biosynthesis	[42]
40	CfEC28	*C. fructicola*	Apple	MdDAHPS1	Chloroplasts	Disrupts primary carbohydrates metabolism	[43]
41	RipAA	*R. solanacearum*	*N. benthamiana*	AtpB	Chloroplasts	Unknown	[44]
42	TGB1	*Alternanthera* mosaic virus	*A. thaliana*	ATP synthase delta, LHCA4, LHB1B2, ATCPISCA, AtpB	Chloroplasts, Mitochondria	Unknown	[45]
43	p50	Tobacco mosaic virus	*N. benthamiana*	NRIP1	Cytoplasm	Triggers NRIP1 relocation to the nucleus via stromules	[47]
44	XopL	*X. campestris* pv. *vesicatoria*	*N. benthamiana*	Unknown	Chloroplasts, Nucleus	Suppresses stromules	[52]
45	PhRXLR-C20	*P. halstedii*	Sunflower	Unknown	Stromules	Unknown	[53]
46	RepA	Citrus chlorotic dwarf-associated virus	*Citrus* spp.	ClMDH	Nucleus^a^	Inhibits ClMDH-induced PCC	[55]
47	VPg	Turnip mosaic virus	*N. benthamiana*, Turnip	NbNdhM	Nucleus^a^	Inhibits NbNdhM-induced PCC	[58]
48	CP	Pepper mild mottle virus	*Capsicum annuum*	CaOMP24	Chloroplasts	Impairs OMP24-induced stromules, PCC and ROS accumulation	[59]

a The interaction between viral proteins and host target proteins is in the nucleus.

b The host target protein CatB is localized at the plasma membrane.

## Pathogen effectors manipulate chloroplast-mediated immunity by affecting ROS homeostasis

ROS, including singlet oxygen species (^1^O_2_), superoxide anion radicals (O_2_^−^), hydroxyl radicals (·OH), and hydrogen peroxide (H_2_O_2_), are high-energy intracellular molecules that play crucial roles in plant defense. In plants, ROS can be generated through two primary pathways: (i) via plasma membrane-localized nicotinamide adenine dinucleotide phosphate (NADPH) oxidase family members and (ii) as a byproduct of photosynthesis in chloroplasts, among which chloroplast-derived ROS (cROS) are particularly significant, as they are persistent ROS, thereby continuously inhibiting pathogen invasion. For example, a more recent study revealed that the chloroplast-localized protein HYPERSENSITIVE TO HIGH LIGHT 1 (HHL1) regulates the effector AvrRpt2-mediated immune activation by precisely modulating cROS homeostasis under different light intensities in *Arabidopsis* [9]. However, to successfully infect plants, many recent breakthroughs have demonstrated that pathogens can suppress the production and accumulation of cROS via effectors, revealing the importance of the homeostasis of cROS in plant–pathogen interactions.

**Figure 1:**
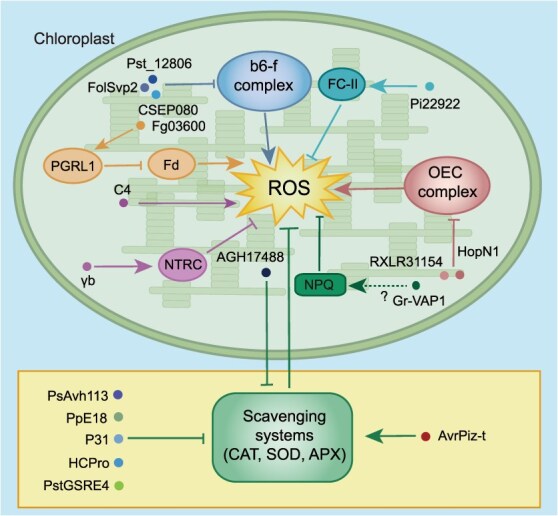
Pathogen effectors hijack chloroplast to maintain pathogenicity. To manipulate plant immunity, many effectors are secreted into the host cell to target the chloroplast and disrupt its functions. For example, the effectors CSEP080, Pst_12806, FolSvp2, RXLR31154, HopN1, Fg03600, and Pi22922 directly target key photosynthetic components, including the b6-f complex, oxygen-evolving complex (OEC), PROTON GRADIENT REGULATION 5-LIKE PROTEIN 1 (PGRL1), and FERROCHELATASE 2 (FC-II), to manipulate redox states and cROS production. Moreover, the effectors γb and Gr-VAP1 disrupt ROS homeostasis by interfering with antioxidant systems and manipulating the function of NPQ, while C4 promotes cROS production for virial signaling transduction. In addition, enzymes in scavenging systems, such as CAT, APX, and SOD are hijacked by many effectors, including PsAvh113, AvrPiz-t, P31, HCPro, PpE18, AGH17488, and PstGSRE4. Fd, ferredoxin.

The generation of cROS often involves key components of the photosynthetic electron transport chain and photosynthetic phosphorylation system and represents a fundamental and early-stage response in plant defense against pathogens. Accordingly, proteins involved in cROS production have become potential targets for pathogen effectors (Table 1). For instance, the *Erysiphe necator* effector CSEP080 specifically targets the host VviB6f, an iron–sulfur subunit of the cytochrome b6-f complex, to promote its accumulation, resulting in promoted photosynthesis and reduced H_2_O_2_ production, which subsequently facilitates pathogen infection in grapevines. These findings suggest that CSEP080 may stabilize the structure of the cytochrome b6-f complex by promoting the accumulation of VviB6f, reducing O_2_·^−^ production caused by electron leakage, thereby decreasing H_2_O_2_ accumulation [10]. Similarly, Pst_12806 from *Puccinia striiformis* f*.* sp. *tritici* (*Pst*) and FolSvp2 from *Fusarium oxysporum* f*.* sp. *lycopersici* (*Fol*) have been shown to hijack the iron–sulfur protein ISP, a putative component of the cytochrome b6-f complex, to attenuate cROS production [11–13], indicating that cROS play essential roles in plant defense and that some pathogens have evolved effectors to manipulate this process. Additionally, the oxygen-evolving complex (OEC) in photosystem II (PS II), which produces cROS, can also be targeted by pathogen effectors. For example, the *Plasmopara viticola* RxLR effector RXLR31154 disrupts the function of photosystem II subunit P (PsbP), and the *Pseudomonas syringae* pv. *tomato* DC3000 (*Pto* DC3000) effector HopN1 induces the proteolysis of PsbQ, both of which mechanistically interfere with the production of cROS [14, 15], indicating that pathogen effectors have mastered the strategy of reducing cROS via direct interaction with cROS-producing proteins, thereby manipulating their functions. Furthermore, effectors attenuate cROS accumulation via interference with metabolic flux in biosynthetic pathways. For instance, Fg03600, an effector from *Fusarium graminearum*, targets wheat PROTON GRADIENT REGULATION 5-LIKE PROTEIN 1 (TaPGRL1) and interferes with its association with ferredoxin (TaFd), thereby suppressing cyclic photosynthetic electron flow [16]. The effector Pi22922 from *Phytophthora infestans* specifically targets the chloroplast protein FERROCHELATASE 2 (StFC-II) and inhibits its degradation in the cytoplasm. Excessive accumulation of StFC-II within the chloroplast disrupts the balance of chlorophyll and heme biosynthesis, ultimately significantly decreasing cROS [17]. These findings indicate that cROS are critical for plant defense and that pathogens have evolved effectors to manipulate the production and accumulation of cROS and subsequently inhibit immunity (Fig. 1).

However, the overaccumulation of ROS also damages plants. To maintain ROS homeostasis, plants have evolved sophisticated intracellular ROS scavenging systems, which include the nonenzyme ascorbate–glutathione system and enzymes, such as superoxide dismutase (SOD), catalase (CAT), and ascorbate peroxidase (APX). Pathogens can also manipulate the ROS scavenging system. For example, CAT contributes to ROS homeostasis by converting H_2_O_2_ into water and oxygen in plants. The *Phytophthora sojae* effector PsAvh113 targets and mediates the degradation of the soybean DRFT1-POLYPEPTIDE B (GmDPB), a transcription factor that regulates the expression of *CAT* [18]. Moreover, to disturb ROS homeostasis, an increasing number of virulence proteins of viruses and effectors from fungi are discovered to bind to CAT and affect its activity [19, 20] (Table 1). Similarly, SOD and APX are also hijacked by effectors [21–23] (Table 1). In addition, pathogen effectors interact with these enzymes across multiple subcellular compartments, including the plasma membrane, cytoplasm, nucleus, and peroxisomes [19–24]. However, the precise mechanisms by which pathogens specifically manipulate cROS homeostasis within chloroplasts remain poorly understood. The nonphotochemical quenching (NPQ) system, which plays a crucial role in photoprotection by mitigating cROS accumulation, may be affected by Gr-VAP1, an apoplastic effector from cyst nematodes [25]. However, the underlying molecular mechanism remains to be addressed. Furthermore, the barley chloroplast-localized copper/zinc superoxide dismutase HvSOD1 contributes to powdery mildew resistance by maintaining the homeostasis of cROS [26]. Further studies are needed to investigate whether effectors target and modulate HvSOD1. Nevertheless, NADPH-DEPENDENT THIOREDOXIN REDUCTASE C (NTRC) balances cROS production and scavenging [27]. It has been reported that the γb protein from barley stripe mosaic virus (BSMV) interacts with NTRC to impair plant antioxidant defenses and facilitate virus replication. Intriguingly, this manipulation appears to be conserved among other chloroplast-replicating viruses, such as lychnis ringspot virus (LRSV) and turnip yellow mosaic virus (TYMV) [28], indicating that interference with cROS homeostasis is a common strategy for viral infection (Fig. 1). Taken together, these findings suggest that the perturbation of chloroplast antioxidant defense systems may represent a conserved pathogenic strategy for pathogen invasion. In addition, further studies are needed to investigate the mechanisms by which plants maintain the homeostasis of ROS in chloroplasts and explore whether pathogens achieve invasion and attack by inhibiting these processes.

## Alteration of the import and function of chloroplast proteins by pathogen effectors

**Figure 2:**
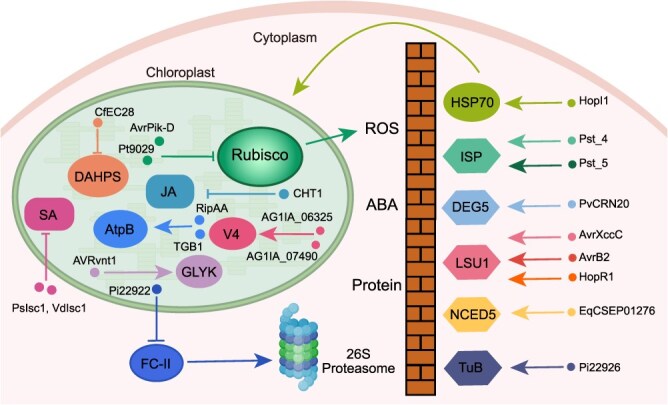
Pathogen effectors affect chloroplast immunity by disturbing chloroplastic protein. The correct import and interaction with partners of chloroplastic proteins are essential for the activation of defense responses. To inhibit plant immunity, pathogen effectors, such as Pst_4, Pst_5, PvCRN20, AvrXccC, AvrB2, HopR1, EqCSEP01276, and Pi22926, affect ABA biosynthesis and reduce cROS by blocking the import of their host target proteins in chloroplasts or altering interactions of their targets with partner proteins. The effector HopI1 translocates its target protein into the chloroplast. While Pi22922 prevents the degradation of its target protein in the chloroplast. Additionally, AG1IA_06325, AG1IA_07490, AvrPik-D, Pt9029, PsIsc1, VdIsc1, CfEC28, TRIPLE GENE BLOCK PROTEIN 1 (TGB1), RipAA, and CHLOROPLAST-TARGETING PROTEIN 1 (CHT1) manipulate chloroplast development and metabolism. AVRvnt1 regulates the post-translational modification of chloroplast proteins. DEG5, DEGP PROTEASE 5; LSU1, LOW SULPHUR UPREGULATED 1; NCED5, 9-CIS-EPOXYCAROTENOID DIOXYGENASE 5.

The compartmentalization of eukaryotic cells necessitates precise protein translocation to target organelles, which is a fundamental requirement for the proper cellular function of proteins. Owing to the fact that many proteins exhibit dual or multiple organelle localizations in plants, pathogen effectors can also manipulate plant immunity by altering the localization of targets, such as the import of chloroplast proteins (Table 1). For instance, Pst_4 and Pst_5, two effectors from *Pst*, impede the chloroplast import of TaISP, thereby suppressing cROS accumulation and promoting immune evasion [13]. Similarly, AvrXccC from *X. campestris* pv. *campestris*, AvrB2 from *P. syringae* pv. *phaseolica*, HopR1 from *P. syringae*, and PvCRN20 from *P. viticola* have been reported to inhibit the accumulation of cROS by blocking their target proteins from entering chloroplasts and disrupting their interactions with chloroplast-localized partners [29, 30] (Table 1). These findings further reveal the complexity of the underlying molecular mechanisms by which pathogens inhibit chloroplast-mediated resistance via interference with protein localization and cROS accumulation. In addition, the *E. quercicola* effector EqCSEP01276 prevents abscisic acid (ABA)-mediated defense in *Hevea brasiliensis* by suppressing the chloroplast import of 9-CIS-EPOXYCAROTENOID DIOXYGENASE 5 (HbNCED5), a key enzyme in ABA biosynthesis [31]. Interestingly, in contrast to the suppression of chloroplast import, the effector HopI1 from *P. syringae* uniquely facilitates the translocation of cytoplasmic Hsp70 into chloroplasts [32], the precise biological significance of which remains unclear. The most likely explanation for this is that pathogens attenuate cytoplasmic Hsp70-mediated immune responses or exploit chloroplast-localized Hsp70 for pathogenicity via the relocalization of Hsp70 to the chloroplast via HopI1. This is similar to the *P. syringae* effector AvrRps4, which is recognized by the NLR protein RESISTANT TO *P. SYRINGAE* 4 (RPS4) in the cytoplasm [33]. However, to escape this recognition, maturely processed AvrRps4 specifically targets chloroplasts, where it displays primary virulence [34]. Taken together, these findings demonstrate that the dynamic relocalization of chloroplast proteins serves as a critical event in plant immunity, which is manipulated by pathogen effectors for pathogenicity (Fig. 2).

Furthermore, pathogens can directly disrupt the function of chloroplast proteins. For instance, the novel pentatricopeptide repeat protein OsV4 regulates chloroplast development in rice and is hijacked by two effectors from *Rhizoctonia solani*, thereby affecting the function of chloroplasts [35, 36] (Fig. 2 and Table 1). The effector AVRvnt1 from *P. infestans* mediates the degradation of GLYCERATE 3-KINASE (GLYK) to counteract its role in basal immunity [37]. Rubisco is an important carboxylase in the C3 carbon reaction of photosynthesis and an indispensable oxygenase in photorespiration. It has been reported that the *Magnaporthe oryzae* effector AvrPik-D suppresses rice immunity by targeting the Rubisco small subunit OsRBCS4 and that the thaumatin-like effector Pt9029 from **P*. triticina* targets the Rubisco activase TaRCA, thereby suppressing host resistance [38, 39]. Furthermore, StTuA/B, two chloroplast elongation factors that strengthen the translation of chloroplast-related proteins, positively regulate disease resistance in potato and *Nicotiana benthamiana*. However, StTuA/B are translated in the cytoplasm and translocated into the chloroplast after being phosphorylated by the MAPK member StMAP3Kβ2. A more recent study revealed that the RxLR effector Pi22926 from *P. infestans* impairs potato fitness and resistance by targeting StTuA/B and suppressing their phosphorylation, which triggers the degradation of StTuA/B in the cytoplasm, thereby reducing their import into chloroplasts and subsequent resistance [40]. These results also reveal the importance of chloroplast proteins in plant immunity, whose accumulation can be suppressed by effectors (Fig. 2). Furthermore, AvrRpt2 regulates cROS homeostasis by activating the phosphorylation of the chloroplast protein HHL1 [9] and Pi22926 reduces the translocation of StTuA/B to chloroplasts by inhibiting their phosphorylation [40], indicating that post-translational modifications (PTMs) are key mechanisms by which effectors manipulate chloroplast functions.

SA is a crucial phytohormone in plant immunity that is biosynthesized in chloroplasts. Pathogens *P. sojae* and *Verticillium dahliae* secrete the isochorismatases PsIsc1 and VdIsc1, respectively, to inhibit SA biosynthesis by redirecting their precursors from the plastid to the cytosol [41]. Furthermore, JA, another central phytohormone in plant defense, plays a pivotal role in mediating resistance against necrotrophic pathogens and herbivorous insects. Notably, key components of JA biosynthesis have emerged as prime targets for pathogen manipulation. For example, the *M. oryzae* effector CHLOROPLAST-TARGETING PROTEIN (MoCHT1) subverts JA-mediated immunity by targeting and stabilizing chloroplast-localized LESION AND LAMINA BENDING (OsLLB), a leucine carboxyl methyltransferase that negatively regulates JA biosynthesis, thereby facilitating rice blast fungal infection [42]. A recent study also revealed that the effector CfEC28 from *Colletotrichum fructicola* interacts with and inhibits the enzymatic activity of the chloroplast-localized 3-DEOXY-D-ARABINOSE-HEPTULONIC ACID-7-PHOSPHATE SYNTHASEs (DAHPSs) in apple, followed by the suppression of DAHPS-mediated secondary metabolite production [43]. Additionally, TRIPLE GENE BLOCK PROTEIN 1 (TGB1) of *Alternanthera* mosaic virus (AltMV) and the type III effector RipAA from *Ralstonia solanacearum* have been shown to interact with the ATPase β subunit AtpB in chloroplasts [44, 45], but the specific underlying mechanisms remain to be addressed in future studies. Taken together, these results indicate that pathogens can affect plant immunity by directly manipulating the stability and function of chloroplast proteins (Fig. 2). However, as NLR proteins are localized mainly in the cytoplasm and at the plasma membrane, it will be interesting to investigate how plants deal with these chloroplast-residing effectors in this arms race. The underlying mechanism holds the potential to engineer elite crops.

## Interference with the communication between the chloroplast and the nucleus by pathogen effectors

**Figure 3:**
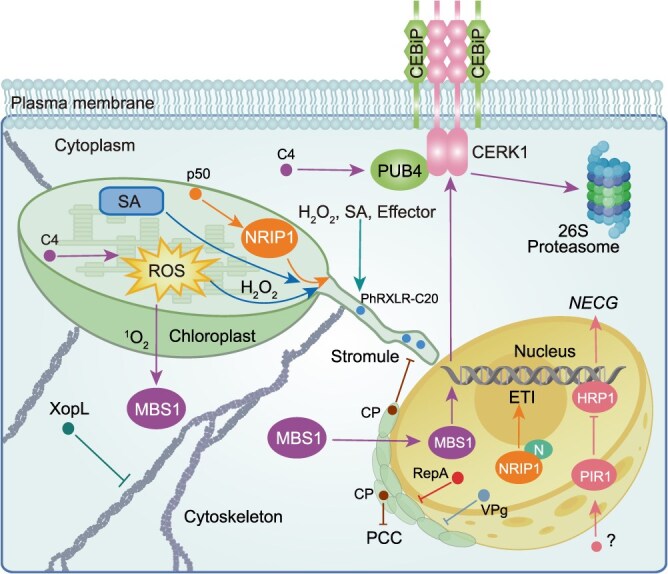
Effectors involved in chloroplast-to-nucleus retrograde signaling pathways. The chloroplast-to-nucleus RS pathway is important in plant immunity, which can be targeted and manipulated by diverse pathogen effectors. For example, chloroplastic C4 (cC4) mediates the translocation of METHYLENE BLUE SENSITIVITY 1 (MBS1) from the cytoplasm to the nucleus via ^1^O_2_ signaling, induces *CHITIN ELICITOR RECEPTOR KINASE 1* (*CERK1*) transcription, and recruits PLANT U-BOX 4 (PUB4) through its membrane variant mC4 to degrade CERK1. P50 triggers NRIP1 relocation to the nucleus via stromules, where NRIP1 is recognized by the nucleotide-binding and leucine-rich repeat receptor (NLR) protein N, thereby triggering ETI. XopL affects the formation of stromules via disruption of the cytoskeleton. PhRXLR-C20 localizes in the stromules. Additionally, RepA, VPg, and coat protein (CP) manipulate host defense by impairing PCC. PIR1, *P. STRIIFORMIS*-INDUCED RING-FINGER PROTEIN 1; HRP1, HISTIDINE-RICH PROTEIN 1.

To efficiently promote disease resistance, accumulating evidence reveals that chloroplasts can communicate with the nucleus and regulate the expression of *NECGs*, termed chloroplast-to-nucleus retrograde signaling (RS), during plant immunity. However, to counteract the RS pathway, a recent study demonstrated that pathogens induce *P. STRIIFORMIS*-INDUCED RING-FINGER PROTEIN 1 (TaPIR1), a ring-finger E3 ubiquitin ligase, to degrade the atypical transcription factor HISTIDINE-RICH PROTEIN 1 (TaHRP1) and suppress the expression of *NECGs* in wheat [46]. Moreover, the chloroplastic effector C4 (cC4) from TGMV induces the production of ^1^O_2_ and promotes the translocation of the cytoplasmic ^1^O_2_ sensor METHYLENE BLUE SENSITIVITY 1 (NbMBS1) to the nucleus, where it activates the expression of *CHITIN ELICITOR RECEPTOR KINASE 1* (*CERK1*), a well-characterized co-receptor that perceives chitin from fungi. However, the membrane-associated mC4, a cC4 variant, recruits NbPUB4 to ubiquitinate and mediate the degradation of CERK1, counteracting its antiviral effects [8]. These findings indicate that pathogen infection will activate information exchanges between the chloroplast and the nucleus (Fig. 3). However, pathogens have also evolved coping strategies to further escape or inhibit plant immunity, revealing the complexity of this arms race in plant–pathogen interactions.

Stromules, highly dynamic tubular protrusions from chloroplasts, are hypothesized structures that serve as channels for transmitting signaling molecules during the chloroplast-to-nucleus RS. It has been shown that PAMPs, SA, and H_2_O_2_ can induce the formation of stromules. Moreover, recent studies have linked the formation of stromules to ETI [47, 48]. For example, the recognition of AvrBS2, an effector from *X. campestris*, by the pepper CNL protein BS2 triggers the formation of stromules in *N. benthamiana* [47]. Similarly, stromule formation has been observed following treatment with various pathogen effectors, including AvrRps4, AvrRpt2, and AvrRpm1 from *P. syringae*, XopQ from *Xanthomonas*, and some effectors from *Melampsora larici-populina* [47, 49, 50]. Furthermore, the calponin homology domain-containing kinesin KINESIN REQUIRED FOR INDUCING STROMULES 1 (KIS1) was discovered to link the chloroplast stroma with immunity by regulating stromule formation. Emerging evidence reveals that KIS-induced stromule formation relies on the core ENHANCED DISEASE SUSCEPTIBILITY 1 (EDS1)/PHYTOALEXIN DEFICIENT 4 (PAD4) module in ETI, thereby establishing a functional link between ETI signaling and chloroplast dynamics [51]. However, the precise mechanism underlying this relationship remains unclear. It has not yet been established whether stromule formation is directly induced by effectors or represents a defense response promoted by plants to enhance immune signaling. Nevertheless, owing to the increase in information exchange between chloroplasts and the nucleus via stromules, it seems that plants actively promote the formation of stromules. Accordingly, chloroplastic target proteins, including N RECEPTOR INTERACTING PROTEIN 1 (NRIP1), are translocated to the nucleus to integrate signals from the chloroplast after treatment with viral proteins, such as the p50 of tobacco mosaic virus (TMV). The translocated NRIP1 protein serves as a guardee and is recognized by the NLR protein N to activate ETI, indicating that plants strengthen chloroplast-to-nuclear RS to quickly respond to pathogen infection [47]. Moreover, stromules may promote the translocation of signaling proteins, such as NRIP1, by increasing the contact area between chloroplasts and the nucleus (Fig. 3), but their specific regulatory factors (e.g. cytoskeletal proteins) and temporal correlation with ETI require further research. Notably, a few effectors have also been reported to target and suppress stromules (Fig. 3). For instance, the *Xanthomonas* effector XopL, which has E3 ubiquitin ligase activity, suppresses stromule formation in *N. benthamiana* [52]. Furthermore, the *P. halstedii* effector PhRXLR-C20 has been identified as a stromule-localized effector [53]. Although whether PhRXLR-C20 plays a negative role in chloroplast-mediated disease resistance needs to be addressed in the future, these findings suggest that multiple pathogen effectors target stromules and that they may function collaboratively in the inhibition of plant disease resistance. In addition, further studies are needed to determine the mechanistic relationship between ETI activation and stromule formation, which will significantly advance our understanding of chloroplast–nucleus communication during defense responses*.*

In addition to stromules, chloroplasts undergo dynamic repositioning and accumulation around the nucleus, known as perinuclear chloroplast clustering (PCC), during the chloroplast-to-nucleus RS [54, 55]. The PCC is generally thought to form a physical barrier around the nucleus in addition to facilitating information exchange between chloroplasts and the nucleus [56]. Several studies indicate that stromule formation often precedes and is required for PCC, potentially serving to guide chloroplast movement via cytoskeletal anchoring and to act as conduits for RS molecules [47, 51, 56]. However, the effector XopQ-induced PCC is not strictly correlated with stromule formation [57], suggesting that PCC and stromule formation represent distinct but functionally collaborative processes during plant immunity. Further investigations are required to clarify the precise functional link and underlying interaction mechanisms between the PCC and stromule formation during chloroplast-to-nucleus RS, which is helpful for fully revealing how chloroplast dynamics contribute to plant immunity. Furthermore, pathogens can counteract host defenses and facilitate infection by disrupting the PCC. For instance, the VPg protein of turnip mosaic virus (TuMV) and the RepA protein of citrus chlorotic dwarf associated virus (CCDaV) recruit their targeted host chloroplastic proteins into the nucleus, thereby impairing the PCC and enhancing viral susceptibility (Fig. 3) [55, 58]. Similarly, the coat protein (CP) of pepper mild mottle virus (PMMoV) disrupts PCC by inhibiting homodimerization of the host protein CHLOROPLAST OUTER MEMBRANE PROTEIN 24 (OMP24) [59]. These findings indicate that the PCC is an effective defensive process employed by plants and that pathogens in turn suppress this process through effector-mediated hijacking. Nevertheless, many underlying precise molecular mechanisms remain to be elucidated, such as whether pathogens inhibit the chloroplast-to-nucleus RS by manipulating the PCC and stromule formation simultaneously and whether there is a common mechanism by which pathogens inhibit PCC, which represents important directions for future research.

## Conclusion and perspectives

The chloroplast is an essential organelle for plant growth and development. In recent years, many advancements have been made toward understanding the role of chloroplasts in plant–pathogen interactions. Moreover, to successfully invade plants, pathogens employ diverse effectors to hijack chloroplast functions, including the suppression of cROS production, interference with the import and function of chloroplast proteins, and manipulation of the chloroplast-to-nucleus RS, thereby weakening plant immunity and promoting infection, which highlights the complexity of the plant–pathogen interaction in this chloroplast battleground. In addition, effectors from different pathogen groups show preferences in targeting chloroplasts: bacterial effectors often inhibit protein transport (e.g. HopI1), fungal effectors focus on interfering with ROS metabolism (e.g. Fg03600), and viral proteins frequently disrupt chloroplast structure (e.g. TGB2). These differences may be related to the pathogen infection mode (intracellular/extracellular).

The intricate interplay between chloroplasts and pathogen effectors opens several promising avenues for future research. One critical area is the elucidation of the underlying molecular mechanisms of the chloroplast-to-nucleus RS. On the one hand, although ROS, metabolites, and translocation proteins have been implicated as potential signaling molecules, the exact pathways and components involved in RS remain poorly understood. On the other hand, how pathogen effectors collaboratively manipulate the RS, especially through the targeting of stromules, will be interesting to be investigated in further studies. Moreover, chloroplasts often maintain close interactions with other organelles, including the endoplasmic reticulum, peroxisomes, and mitochondria, through membrane contact sites and shared signaling molecules during plant immunity [60]. Future studies need to explore whether pathogen effectors directly affect chloroplast heterogeneity, such as size, positioning, and functional differentiation, which ultimately interferes with the allocation of chloroplast signals and influences interactions between chloroplasts and other organelles, thereby impairing plant immune responses. In addition, PTMs, such as phosphorylation, ubiquitination, and acetylation, are crucial for the functional regulation of proteins [8, 37, 42]. Many effectors have been demonstrated to alter plant immunity by affecting the PTMs of target proteins. However, the manipulation of PTMs on chloroplast proteins by pathogen effectors remains to be addressed. Furthermore, plants employ NLR proteins to recognize effectors and initiate a much stronger ETI response that further restricts the growth and spread of invading pathogens. One fatal issue is that NLR proteins are often localized in the cytoplasm or at the plasma membrane, whereas chloroplast-localized effectors are not localized in the same subcellular compartments of NLRs. Moreover, multiple effectors may coordinately target chloroplasts to manipulate host immunity. Therefore, the potential coordinating mechanism of multiple effectors and the functional link between NLRs and chloroplast-localized effectors remain to be addressed in the future. One most possible research direction is that NLRs may recognize chloroplast-localized effectors through PTM mechanisms. Additionally, the coordination among pathogen effectors may influence this recognition, which should also be an important area to be explored in the future. Answers to these questions are helpful for fully understanding the role of chloroplasts in plant–pathogen interactions, revealing comprehensive perspective on the arms race between plants and pathogens, and providing new insights into the engineering of elite resistant crops.

## Data Availability

The data presented in this study are available in the article.
